# The Outcome of Phagocytic Cell Division with Infectious Cargo Depends on Single Phagosome Formation

**DOI:** 10.1371/journal.pone.0003219

**Published:** 2008-09-16

**Authors:** Yong Luo, Mauricio Alvarez, Lingchuan Xia, Arturo Casadevall

**Affiliations:** 1 Department of Microbiology and Immunology, Albert Einstein College of Medicine, Bronx, New York, United States of America; 2 Jiaomen 13th Yuan, Beijing, People's Republic of China; 3 Department of Medicine, Albert Einstein College of Medicine, Bronx, New York, United States of America; University of Birmingham, United Kingdom

## Abstract

Given that macrophages can proliferate and that certain microbes survive inside phagocytic cells, the question arises as to the post-mitotic distribution of microbial cargo. Using macrophage-like cells we evaluated the post-mitotic distribution of intracellular *Cryptococcus* yeasts and polystyrene beads by comparing experimental data to a stochastic model. For beads, the post-mitotic distribution was that expected from chance alone. However, for yeast cells the post-mitotic distribution was unequal, implying preferential sorting to one daughter cell. This mechanism for unequal distribution was phagosomal fusion, which effectively reduced the intracellular particle number. Hence, post-mitotic intracellular particle distribution is stochastic, unless microbial and/or host factors promote unequal distribution into daughter cells. In our system unequal cargo distribution appeared to benefit the microbe by promoting host cell exocytosis. Post-mitotic infectious cargo distribution is a new parameter to consider in the study of intracellular pathogens since it could potentially define the outcome of phagocytic-microbial interactions.

## Introduction

Until relatively recently it was commonly believed that macrophages were in a post-mitotic state [1], [2], [3]. In this scenario, dead and damaged macrophages in tissue are replenished by an influx of blood monocytes that then differentiate into tissue macrophages [4], [5]. However, there is now considerable evidence that tissue-derived macrophages retain a capacity to proliferate under suitable conditions. Macrophages can extensively proliferate *in vitro* when stimulated with macrophage growth factor, which is present in most tissues at concentrations sufficient to sustain cell division [6], [7], [8]. In addition, a significant proportion of peritoneal macrophages and macrophages in local tissue appear to undergo mitosis *in vivo,* especially during inflammatory conditions [9], [10], [11], [12], [13], [14], [15], [16], [17], [18]. Recently, our group has shown that opsonic phagocytosis can stimulate cell cycle progression [19], through an ERK signaling mechanism (data presented at Experimental Biology 2008 meeting at San Diego, CA, Abstract #LB545). These findings suggest that local proliferation of macrophages in infected tissue could provide an alternative mechanism for the replenishment of tissue macrophages upon damage by infection.

Given that macrophages can undergo cell division locally in tissue under various conditions and that certain microbes can survive inside phagocytic cells, the question arises as to the fate of intracellular pathogens when the original phagocytic cell divides into two daughter cells. To our knowledge, this important question has not been systematically studied. In fact, consideration of possible outcomes reveals that macrophage cell division is potentially a double-edged sword from the viewpoint of host defense mechanisms. On one hand, cell division would double the number of phagocytic cells, an event that could conceivably benefit the host by increasing the number of effector cells at the site of infection. On the other hand, division of cells infected with a live microbe that survives intracellularly could also lead to a doubling of infected cells, an event that could facilitate the dissemination of infection and potentially harm the host. Certainly, the benefit or debit to the host of infected macrophage replication would also depend on the distribution of microbes among infected cells and on the type of pathogenic microbes. In situations where the entire microbial load would segregate to one cell, there would be a new uninfected cell generated for host defense or for new microbial invasion and proliferation, depending on the circumstances. Hence, the realization that macrophages can replicate combined with the ability of certain microbes to replicate intracellularly poses several new problems for innate immunity and microbial pathogenesis.

*Cryptococcus neoformans* is a major cause of life-threatening infections such as pulmonary cryptococcosis and meningoencephalitis in patients with impaired immunity. Macrophages play a vital role in cryptococcal pathogenesis [20], [21]. *C. neoformans* is a facultative intracellular pathogen in macrophages. Alveolar macrophages containing intracellular *C. neoformans* are the important component in dormant form of *C. neoformans* infection which as the potential for later reactivation if the host becomes immunocompromised [22], [23], [24]. The role of macrophages in defense against *C. neoformans* appears to depend on the host species such that pulmonary macrophage depletion is associated with increased susceptibility and resistance, in rats and mice, respectively [25]. *C. neoformans* has a unique and remarkable intracellular pathogenic strategy that includes replication inside macrophages, accumulation of cytoplasm polysaccharide-containing vesicles and induction of phagosome leakiness [22], [23]. Recently, a novel phenomenon known as phagosomal extrusion has been described, whereby *C. neoformans* can escape from the macrophage host, with survival of both yeast and host cells [26], [27].

In the present study, we evaluated the distribution of intracellular yeasts into daughter cells following mitosis of macrophage-like cells. The data suggested that the outcome of particle distribution is a function of the intracellular cargo number, with inert particles and certain *C. neoformans* strains having a stochastic outcome whereas for others, the distribution was unequal and not determined by chance alone. The outcome of ingested particle distribution depends on the single phagosome formation, thus implying that phagosomal fusion events can have a dominant effect on intracellular particle distribution following phagocytic cell division.

## Results

### Modeling of post-mitotic intracellular particle distribution

**Figure 1:**
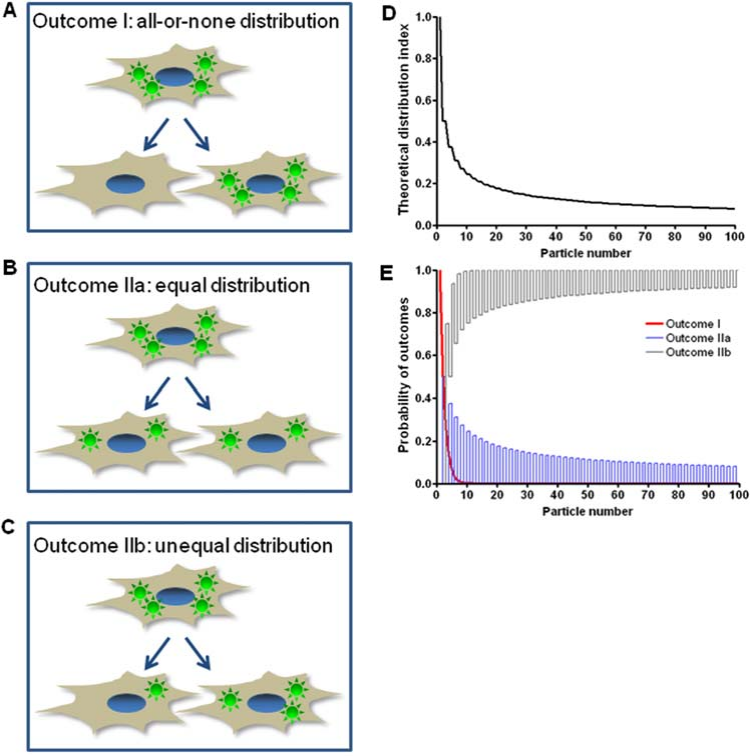
The possible post-mitotic outcomes of intracellular particle distribution and modeling of post-mitotic intracellular particle distribution. (A) Schematic diagram of Outcome I: all the intracellular particles in the mother cell are distributed into one of the daughter cells with a clean daughter cell generated after cell division (all-or-none distribution). (B and C) Schematic diagram of Outcome II: intracellular yeast cells in the mother cell are distributed equally (B) or unequally (C) into both daughter cells after cell division (equal or unequal distribution). (D) Theoretical distribution index (DI_T_) for various numbers of intracellular particles calculated from Equation (4), with the aid of a computer program, assuming that the intracellular particles were stochastically distributed into two daughter cells during cell division. DI_T_ for *n* = 1–100 were plotted in this Figure. (E) Probability of Outcome I, IIa and IIb (P_I_, P_IIa_ and P_IIb_) of various numbers of intracellular particles calculated from Equations (5)–(7), with the aid of a computer program, assuming that the intracellular particles were stochastically distributed into two daughter cells during cell division. P_I_, P_IIa_ and P_IIb_ for *n* = 1–100 were plotted in this Figure.

Following phagocytosis of yeasts, a significant portion of J774.16 macrophage-like cells undergo cell division. This effect probably reflects the phenomenon of cell cycle progression following phagocytosis that we have described earlier [19]. To analyze the outcome of intracellular yeast distribution after phagocytic cell division we considered that the default outcome was stochastic, with the distribution of ingested particles or microbes after cell division reflecting chance alone. There are two possible outcomes to the division of an infected cell (Figure 1A, B and C). Outcome I occurs when all the intracellular particles in the mother cell are distributed into one of the daughter cells, a process that produces one infected and one with a clean daughter cell (Figure 1A). Outcome II occurs when the yeast cells in the mother cell are distributed to both daughter cells after cell division. Furthermore, Outcome II can be subdivided into two conditions: the intracellular yeast cells are equally distributed into both daughter cells (Outcome IIa, Figure 1B) and unequally distributed into both daughter cells (Outcome IIb, Figure 1C). Conceivably, these two conditions are relevant to microbial pathogenesis because Outcome IIa leaves both daughter cells with half the number of ingested yeasts and thus could allow cellular microbicidal mechanisms to overcome the microbial infection while in Outcome IIb, one daughter cell is left with larger microbial load compared to the other.

To analyze the distribution of intracellular particles during macrophage-like cell division quantitatively, the skewness of numerical difference of intracellular particles between two daughter cells was measured by the distribution index (DI) which was defined as following. The number of intracellular particles in mother cell is *n*. During cell division, there are various outcomes for intracellular particle distribution between the two daughter cells. If the number of intracellular particles which are distributed into Daughter cell 1 is *x*, the number of intracellular particles which are distributed into Daughter cell 2 is *n-x*. The DI was defined as
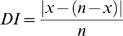
(1)


Based on the stochastic outcome we developed an algorithm to model the intracellular particle distribution after cell division. The number of intracellular particles in any given mother cell is *n*. During cell division, the intracellular particles are assumed to be distributed stochastically into two daughter cells. If the number of intracellular particles which are distributed into Daughter cell 1 is *x*, the number of intracellular particles which are distributed into Daughter cell 2 is *n-x*. The probability of this given event to occur is *y*, which can be calculated by the equation
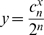
(2)Note that 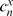 is the number of possible events to distribute *x* intracellular particles into one daughter cell if total *n* intracellular particles are distributed and 2*^n^* is the total number of possible events to distribute *n* intracellular particles into two daughter cells.

The DI for this given event (*z*) is the probability multiplied by the numerical difference of the intracellular particles between two daughter cells
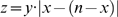
(3)The theoretical DI (DI_T_) for all possible distribution events of *n* intracellular particles is
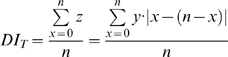
(4)


Stochastic probabilities of Outcome I, IIa and IIb events described above, which also vary as the function of particle number, were calculated based on Equation (2). For probability of Outcome I (P_I_), either Daughter cell 1 has all the particles (*x = n*) or Daughter cell 2 has all the particles (*x* = 0)
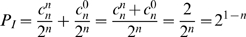
(5)


The probabilities of Outcome IIa (P_IIa_) or Outcome IIb (P_IIb_) depend on whether the number of particles in mother cells is even or odd. It is impossible to have Outcome IIa or Outcome IIb events if the number of particles in the mother cells is odd or even, respectively. Therefore, P_IIa_ equals 0 and P_IIb_ equals 1 when *n* is odd whereas P_IIa_ equals 1 and P_IIb_ equals 0 when *n* is even. Other than in these exclusionary conditions,
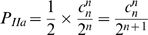
(6)

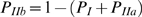
(7)


To handle the computational complexity that results with an increasing number of ingested particles (*n*), we wrote two programs using sun java jdk1.6 to calculate DI_T_ and P_I_, P_IIa_ and P_IIb_ based on Equation (4)–(7) (Data Set S1, S2 and S3 were the java programs for calculation of DI_T_, calculation of P_I_, P_IIa_ and P_IIb_ and factorial calculation respectively). Java standard library functions were used for coding. Since floating-point numbers may lose precision, the programs can only be used when *n*≤1000. Given macrophage-like cells in our system rarely ingested particles more than 50, the calculated numbers were sufficient for our comparison and normalization purposes.

Using the above formalisms we calculated the DI_T_ and P for situations up to *n* = 100 and plotted it as a function of ingested particle number (Figure 1D, E and Data Set S4, S5, which were calculated results of DI_T_ and P_I_, P_IIa_ and P_IIb_ respectively). When there is only one particle per cell, the distribution will always be an Outcome I event, which has a DI_T_ of 1. The DI_T_ drops as a function of particle number as the particles per cell increase (Figure 1D). P_I_ drops rapidly as a function of particle number when the particle per cell increase, such that an Outcome I event becomes extremely unlikely if the particle number is more than 10. P_IIa_ decreases and approach to 0, as the particle number increases given the particle number is not odd. Conversely, P_IIb_ increases and approach 1 as particle number increases given the particle number is not even (Figure 1E). Hence, if the distribution of ingested particles is determined by chance alone the likelihood that a clean daughter cell emerges from cell division is dramatically reduced as a function of the number of ingested particles. This analysis provided a reference point for the comparison and interpretation of the biological data obtained with phagocytic cells.

### Post-mitotic distribution of intracellular yeasts

**Figure 2:**
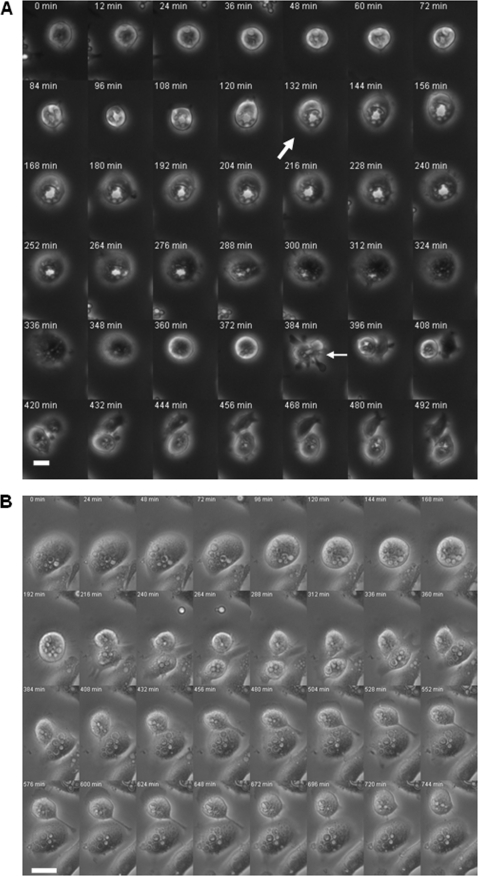
Outcome I and II of post-mitotic intracellular particle distribution. (A) Post-mitotic Outcome I of intracellular particle distribution. Images showing J774.16 cells infected with HK Cap67 undergoing cell division and sorting the intracellular yeasts into one of the two daughter cells. Frames are labeled according to the start of the imaging process, which is approximately 1 h after phagocytosis of yeasts was initiated. The thick arrow indicated single giant phagosome formation caused by phagosomal fusion. The thin arrow indicated macrophage-like cell division. Images were collected at 20×. Bar, 10 µm. (B) Post-mitotic Outcome II of intracellular particle distribution. Images showing J774.16 cells infected with *C. neoformans* strain 24067 undergoing cell division and sorting the intracellular in both daughter cells. Frames are labeled according to the start of the imaging process, which is approximately 1 h after phagocytosis of yeasts was initiated. The arrow indicated macrophage cell division. Images were collected at 20×. Bar, 10 µm.

We measured the distribution of intracellular yeasts into the daughter cells using microscopic time-lapse imaging after phagocytosis of three different *C. neoformans* strains, 24067, H99 and Cap67, which is an acapsular mutant. For a comparison, a different *Cryptococcus* spp., *Cryptococcus gattii* was also used in our experiments. In addition, we investigated the distribution of inert polystyrene beads ingested by macrophage-like cells as the control. To explore whether microbial factors affected the measured outcomes, we also evaluated heat-killed H99 strain (HK H99), one H99 strain deficient in phospholipase B (H99 PLB1), heat-killed Cap67 (HK Cap67), Cap67 coated with polysaccharide derived from H99 (Cap67+PS) and heat-killed *C. gattii* (HK *C. gattii*). As predicted, both Outcomes I and II events were observed during cell division of macrophage-like cells containing yeasts or beads (Figure 2A and Movie S1, Figure 2B and Movie S2, respectively). We noted that formation of a single giant phagosome often preceded Outcome I type cell division. Before cell division, phagosomes with intracellular yeasts often fused together, forming a single giant phagosome and effectively reducing intracellular particle number (*n*) to 1. The integrity of the giant phagosome was maintained during cell division and consequently the phagosome was sorted into one of the daughter cells after cell division. Interestingly, the likelihood of single giant phagosome formation was particularly high when phagocytic cells contained *C. neoformans* strains H99 PLB1 mutant, Cap67 and *C. gattii* yeast cells (Figure 2A and Movie S1). In contrast, the single giant phagosome formation was rarely seen in Outcome II type cell division when phagocytic cells contained polystyrene beads or *C. neoformans* strain 24067 (Figure 2B and Movie S2).

In addition, similar distribution patterns were observed in yeast-infected tissue-derived macrophages during cell division in culture (data not shown). However, the frequencies of cell division events for tissue-derived macrophages were much lower than observed with macrophage-like cell lines and consequently we focused our analysis on data derived from J774.16 macrophage-like cells. These macrophage-like cells faithfully reproduce the interaction of yeasts with macrophages but have the advantage of reproducing every 12 h [19] and thus provide an extremely useful system for studying the outcome of phagocytic cell division in the setting of intracellular ingested cargo.

### Comparison of experimental and theoretical post-mitotic particle distribution

**Figure 3:**
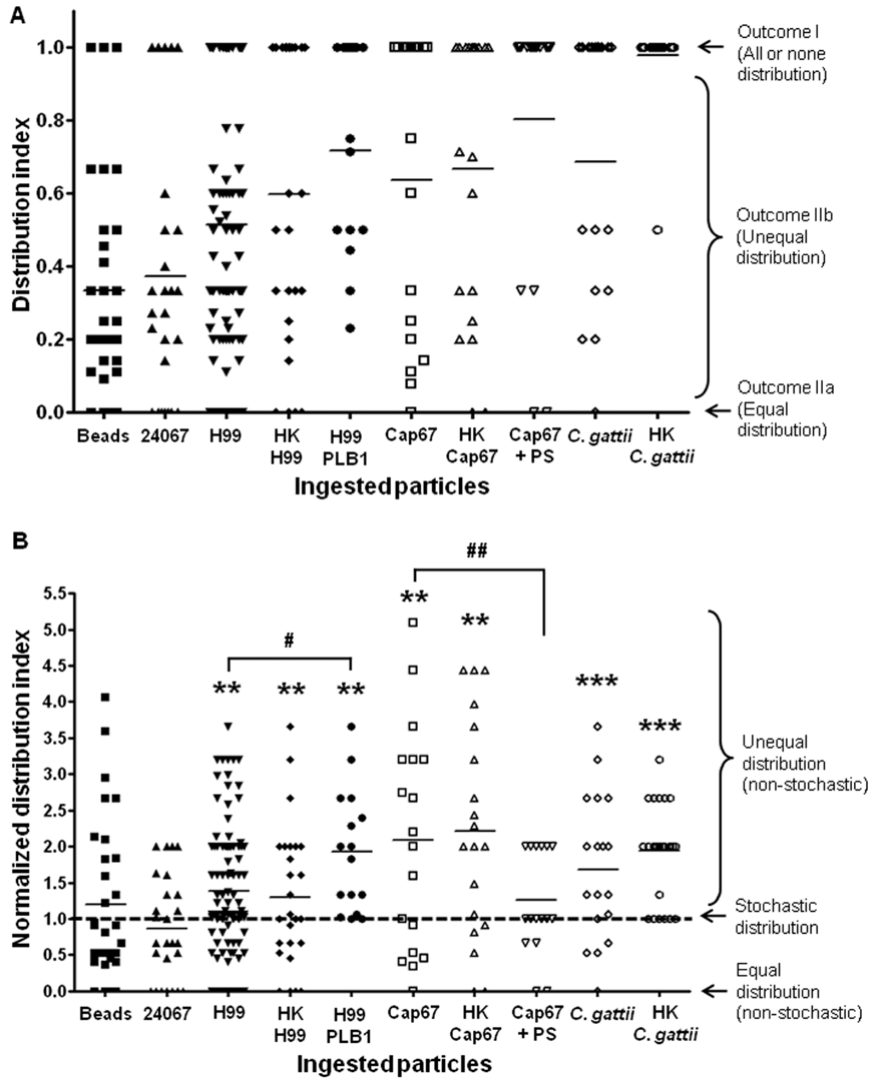
Comparison of experimental and theoretical post-mitotic particle distribution. (A) Post-mitotic DI of intracellular particle distribution. DI for *C. neoformans* strains 24067, H99, heat-killed H99 (HK H99), H99 phospolipase mutant (H99 PLB1), Cap67, heat-killed Cap67 (HK Cap67), Cap67 coated with polysaccharide (Cap67+PS), *C. gattii* or heat-killed *C. gattii* (HK *C. gattii*). The horizontal lines denoted the means of calculated DI, which were not immediately comparable between the groups as described in the Results. The data were collected from three or more independent experiments for every condition. In every experiment, movies were recorded for approximately 24 h with 300–400 cells in the microscopic field from which the macrophage-like cells underwent cell division with intracellular particles were selected for analysis. (B) Normalized post-mitotic DI by stochastic modeling of intracellular particle distribution. To eliminate the inaccuracy introduced by numerical variances of intracellular particles, the normalized DI was calculated as described in the Results. The distribution of intracellular particles was stochastic if the normalized DI equaled to one (dash line). While normalized DI were either larger or smaller than 1, the distribution of intracellular particles into daughter macrophage-like cells was non-stochastic and was skewed to unequal distribution or equal distribution, respectively. One-way ANOVA test revealed significant variances between the groups (*p*<0.0001). Newman-Keuls multiple comparison test revealed significant difference between H99 and H99 PLB1 (^#^ *p*<0.05), Cap67 and Cap67+PS (^##^ *p*<0.01). One sample *t* test revealed that post-mitotic distribution of most yeasts had significantly larger DI than 1 which denoted stochastic distribution (dash line) (**p<0.01, ***p<0.001).

To quantify the distribution of intracellular particles into daughter cells after division of macrophage-like cells the outcome data was presented as the DI. In Outcome I, the DI equals 1. In Outcome II, the DI is between 0 and 1 for any unequal distribution (Outcome IIb) whereas the DI for equal distribution (Outcome IIa) would equal to 0. The DI and the distribution outcomes of yeasts and polystyrene beads were shown in Figure 3A. The measured experimental P_I_, P_IIa_ and P_IIb_, which were calculated by dividing outcome events by total events, were shown in Table 1. For comparison, the theoretical probabilities of the outcomes for yeasts and beads were calculated according to the number of ingested particles using the plot in Figure 1E. For polystyrene beads, the distribution of particles into daughter cells was that expected by chance alone as no significant difference was revealed between the experimental data and the theoretical calculation. For the intracellular yeasts, P_I_ for all strains except *C. neoformans* strain 24067 were significantly higher than expected by chance alone. Interestingly, large differences of probabilities between experimental data and theoretical calculation observed when the intracellular particles were H99 PLB1 mutant, Cap67, HK Cap67, *C. gattii* and HK *C. gattii*. These results suggest that microbial-mediated actions, possibly via phospholipase or polysaccharide, can skew the distribution of ingested particles into one of the daughter cells during cell division. We also noted that these yeasts also had particularly high single giant phagosome formation before macrophage-like cell division as described above.

**Table 1:** Experimental and theoretical probabilities of distribution outcomes

%	Experimental probability			Theoretical probability		
Outcome	I	IIa	IIb	I	IIa	IIb
**Beads**	10	13	77	6	15	79
**24067**	19	27	54	19	17	64
**H99**	29*	10	61	16	17	67
**HK H99**	44*	19	37	30	18	52
**H99 PLB1**	44* #	0	56	20	21	59
**Cap67**	50**	6	44	12	14	74
**HK Cap67**	50***	10	40	9	19	72
**Cap67+PS**	76* §	12	12	59	15	26
***C. gattii***	53*	5	42	24	27	49
**HK** ***C. gattii***	96***	0	4	43	17	40

Probabilities of distribution outcomes observed in the experiments (Figure 3A) were calculated. Theoretical probabilities of distribution outcomes were calculated from the plot in Figure 2B according to the number of ingested particles. χ*^2^* analysis was used to compare P_I_ between different groups.

* indicates significant difference between experimental and theoretical probabilities (^*^ *p*<0.05, ^**^ *p*<0.01, ^***^ *p*<0.001).

# indicates significant difference between H99 PLB1 mutant and it parent strain H99 (^#^ *p*<0.05).

§ indicates significant difference between Cap67 coated with H99 polysaccharide and Cap67 (^§^ *p*<0.05).

We originally intended to compare DI between macrophage-like cells dividing with intracellular beads and yeasts. However, the means of the calculated DI (horizontal lines in Figure 3B) were not immediately comparable between these groups because the numbers of intracellular particles ingested by macrophage-like cells before cell division event differed between groups. Given that the stochastic outcome of the DI can vary as a function of the number of ingested particles, we resorted to comparing the experimental outcomes observed to the DI_T_ predicted by the stochastic modeling described previously (Figure 1D and Data Set S4, S5). Consequently, the normalized DI was calculated by dividing the DI obtained from the experiments (showed as dots in Figure 3A) with the corresponding DI_T_ of the same number of intracellular particles (Figure 3B). Using this normalization approach, we eliminated the inaccuracy introduced by numerical variances of intracellular particles among groups. By this analysis, the distribution of intracellular particles was stochastic if the normalized DI equaled 1 (dash line in Figure 3B). While normalized DI for yeasts and beads were either larger or smaller than 1, the distribution of intracellular particles into daughter macrophage-like cells was non-stochastic and skewed towards unequal distribution or equal distribution, respectively. Statistical analysis showed that the normalized DI were significantly larger than 1 for all the yeasts except *C. neoformans* strain 24067, Cap67+PS and beads. Interestingly, the H99 PLB1 mutant had significantly higher DI than its parental strain H99. When the acapsular mutant Cap67 was coated with polysaccharide from strain H99, this strain had a similar DI as that of H99.

### Single phagosome formation as a mechanism for non-stochastic intracellular particle distribution

**Figure 4:**
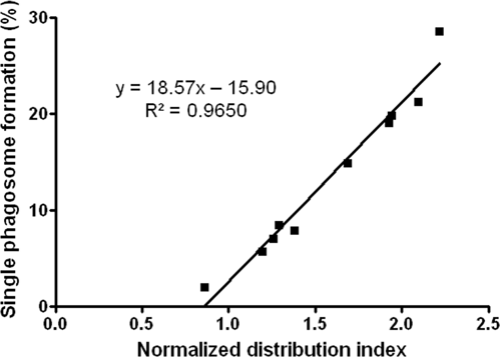
Linear correlation of percentage of macrophage-like cells with single phagosome formation versus the normalized DI. The percentages of cells demonstrating single phagosome formation in all the experiments were calculated and plotted versus the normalized DI. Statistical analysis revealed a strong linear correlation. The best-fit line intersected the X-axis near a normalized distribution index of 1, which denotes a stochastic distribution in situations where there is no phagosomal fusion.

Phagosomes containing intracellular *Cryptococcus* yeasts can fuse, leading to formation of giant phagosomes in macrophages [26], [28]. Hence, we suspected that single giant phagosome formation played an important role in the post-mitotic distribution of intracellular particles. To test this possibility, we counted single phagosome formation events in all experiments to determine the percentage of yeast containing cells demonstrating single phagosome formation. Plotting the percentage of cells forming single phagosome versus the normalized DI revealed a strong linear correlation (Figure 4). Interestingly, the best-fit line intersected the X-axis near a normalized DI of 1, implying a stochastic distribution in situations where there was no single phagosome formation.

### Biological consequences following Outcome I and II of intracellular particle distribution during macrophage-like cell mitosis

**Figure 5:**
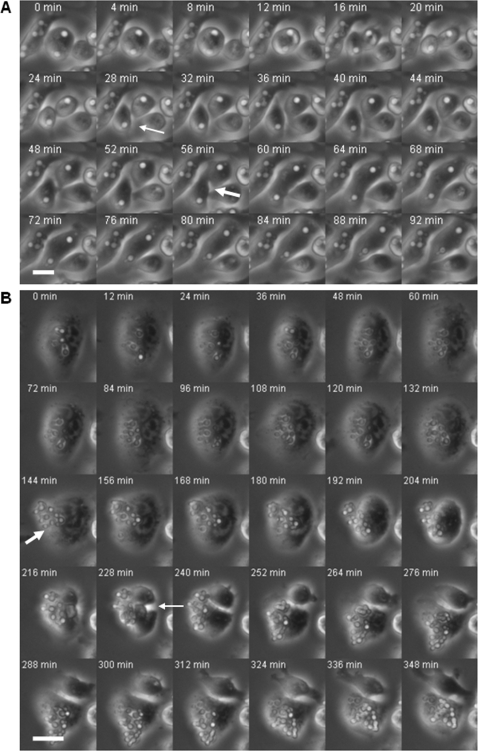
Extrusion of intracellular yeasts during mitosis of macrophage-like cells and fusion of two daughter macrophage cells with intracellular yeasts post mitosis. (A) Fusion of two daughter macrophage cells with intracellular yeasts post mitosis. Frames are labeled according to the start of the imaging process, which is approximately 1 h after phagocytosis of *C. neoformans* strain H99 was initiated. The thick arrow indicated the fusion of two daughter cells after division and the thin arrow indicated macrophage-like division. Images were collected at 20×. Bar, 10 µm. (B) Extrusion of intracellular yeasts during mitosis of macrophage-like cells. After phagocytosis of *C. neoformans*, some macrophage-like cells undergoing cell division extruded intracellular yeasts either before or after the cell round-up, a morphological change that indicates the initiation of cell division (time zero). Frames are labeled according to the start of the imaging process, which is approximately an hour after phagocytosis of *C. gattii* was initiated. The thick arrow indicated the extrusion of intracellular *C. neoformans* and the thin arrow indicated macrophage-like cell division. Images were collected at 20×. Bar, 10 µm.

To investigate the biological consequences of intracellular particle distribution, the two daughter cells were continuously observed by time-lapse microscopy after cell division. Four types of consequences were observed and quantified following Outcome I and II events: cell survival, cell death by lysis, phagosomal extrusion and daughter cell fusion (Table 2 and 3). The overwhelming majority of daughter cells emerged from mitosis alive as indicated by movement irrespective of whether they had intracellular cargo. Lysis of the cells caused by intracellular yeasts was not commonly observed during our experimental time frame. We occasionally observed the fusion of two daughter cells with intracellular yeasts after the cell division was completed (Figure 5A and Movie S3). This phenomenon was not observed with uninfected macrophage-like cells or macrophage-like cells containing intracellular beads. The fusion phenomenon was relatively rare, but its occurrence is noteworthy because it implies an altered cell membrane state for cells with ingested yeasts. In addition, phagosomal extrusion of intracellular yeasts was commonly observed before as well as after mitosis and intracellular yeast distribution (Figure 5B and Movie S4). This phenomenon indicates that phagosomal extrusion does not damage the host cell sufficiently to prevent cell division. The extrusion was more commonly seen with intracellular *C. gattii*, less commonly seen with *C. neoformans* strains. No extrusion was observed with macrophage-like cells harboring intracellular beads. These results were consistent with the previous report [26]. Mitosis resulting in Outcome I was associated with more phagosomal extrusion events. Interestingly, compared to the phagosomal extrusion rates reported previously under no mitosis conditions [26], the daughter cell with full cargo load post Outcome I distribution had significantly higher extrusion rates with intracellular *C. neoformans* strains Cap67, HK Cap67+PS, H99 and HK C. *gattii*, suggesting that post-mitotic host cells were more likely to demonstrate microbe exit events. The extrusion rates were similar if intracellular yeasts were *C. neoformans* strain 24067 and *C. gattii*. In the contrast, extrusion rates post Outcome II distribution were lower than those reported under no mitosis conditions. This implied that Outcome I events may predispose intracellular yeasts for extrusion whereas Outcome II events reduce the likelihood of phagosomal extrusion.

**Table 2:** Rates of the four consequences observed after Outcome I distribution

%	Daughter cell 1				Daughter cell 2			
Particles	MΦ live	MΦ lysed	particle extruded	MΦ fused	MΦ live	MΦ lysed	particle extruded	MΦ fused
**Beads**	100	0	0	0	100	0	0	0
**24067**	75	0	0	25	50	0	25	25
**H99**	100	0	0	0	57	0	43**	0
**HK H99**	100	0	0	0	59	0	41*	0
**H99 PLB1**	100	0	0	0	29	0	71*	0
**Cap67**	100	0	0	0	56	0	44*	0
**HK Cap67**	100	0	0	0	44	0	56*	0
**Cap67+PS**	93	0	0	7	57	7	29*	7
***C. gattii***	100	0	0	0	50	0	50*	0
**HK ** ***C. gattii***	96	0	0	4	70	4	22*	4

**Table 3:** Rates of the four consequences observed after Outcome II distribution

%	Daughter cell 1				Daughter cell 2			
Particles	MΦ live	MΦ lysed	particle extruded	MΦ fused	MΦ live	MΦ lysed	particle extruded	MΦ fused
**Beads**	100	0	0	0	100	0	0	0
**24067**	95	0	0	5	82	0	14	4
**H99**	87	0	6	7	78	0	14	8
**HK H99**	100	0	0	0	100	0	0	0
**H99 PLB1**	67	0	33	0	56	0	44	0
**Cap67**	89	11	0	0	67	11	22	0
**HK Cap67**	90	0	0	10	80	0	10	10
**Cap67+PS**	100	0	0	0	100	0	0	0
***C. gattii***	63	0	25	12	50	0	37	13
**HK ** ***C. gattii***	100	0	0	0	100	0	0	0

Time-lapse microscopy recordings were performed after phagocytosis of different particles by macrophage-like cells. Four different consequences including conditions as daughter cells were live, lysed, fused or intracellular yeasts were extruded were observed post Outcome I and II distribution (A and B respectively). The percentages of events observed were shown. Daughter cell 1 and 2 generated by Outcome I were the cells without intracellular yeasts and with full load of intracellular yeasts, respectively. Daughter cell 1 and 2 generated by Outcome II were the cells with small number of yeasts and with large number of intracellular yeasts, respectively. χ*^2^* analysis revealed significant higher rates of phagosome extrusion occurred post Outcome I distribution if intracellular particles were yeasts except *C. neoformans* strain 24067 and beads compared to that of post Outcome II distribution (^*^ *p*<0.5, ^**^ *p*<0.01).

## Discussion

The predicted distribution outcomes for mitotic phagocytic cells carrying ingested particles were each experimentally observed in this study. In Outcome I, intracellular yeasts are quarantined within one of the daughter macrophage-like cells during mitosis, leaving the other daughter cell microbe- or particle-free. In Outcome II, intracellular yeasts were unequally or equally distributed into daughter macrophage-like cells. Intracellular yeast distribution via Outcome I may be beneficial to the host since it can cure a cell of infection by generating one clean cell. In contrast, equal or unequal distribution corresponding to Outcomes II produced two infected macrophages that could, in theory, perpetuate the infection and facilitate dissemination in conditions where the host cell does not kill the microbe. Our data showed that a significantly large percentage of macrophage-like cells harboring intracellular *C. gattii* and acapsular *C. neoformans* strain had Outcome I distribution, compared to those with intracellular beads and capsular *C. neoformans* strains. Interestingly, Outcome I distribution has been noted in at least one other system. Almost two decades ago, the similar phenomenon was observed in the distribution of intracellular *Coxiella burnetti* during macrophage cell division [29], [30]. In the case of *C. burnetti*, unequal distribution resulted from the formation of a single large phagosome that necessarily produced an Outcome I event after cell division. Similarly, our study indicated that the mechanism responsible for Outcome I in the case of yeasts appears to be a phagosome fusion event that produces a single large phagosome sortable between the two daughter cells.

Our analysis was limited to macrophage-like cells and we acknowledge the limitations inherent in using immortalized cell lines *in vitro*. Nevertheless, J774.16 macrophage-like cell line are a good system because they faithfully reproduce the essential events required for this process, namely phagocytosis, phagosome fusion, intracellular replication, and phagosome extrusion that have also been observed with tissue derived macrophages [19], [23], [26]. Furthermore, we did observe division of primary macrophages containing ingested particles in this study but the frequency of such events *in vitro* was too low for a statistically significant analysis. The fact that J774.16 macrophage-like cells reproduce many of the effects observed with primary macrophages and that similar observations were made with primary macrophages provides confidence for assuming that similar outcomes can be expected for tissue macrophages that are induced to replicate during infection.

Outcome I type phagocytic cell division generates a new clean phagocytic cell for host defense while confining the infection to another cell. Given that Outcome I generates a clean daughter cell, one might surmise that this is the best outcome for the host. However, from the microbial perspective, Outcome I events also generate a new host cell for infection and potentially doom the infected daughter cell by burdening it with a higher intracellular microbial load. Furthermore, Outcome I events would not necessarily be best for the host if dividing the infectious cargo into two cells would reduce intracellular microbial burden and allow the host cell to kill or inhibit the microbe in the intracellular space. Hence, it is not clear whether Outcome I events are optimal for the host or the microbe and it is likely the beneficiary of this event will depend on the specific type of host-microbe interaction and the survivability of the microbe in the intracellular environment. From a teleological perspective, it is likely that the all-or-none nature of Outcome I events could be a decisive event on the outcome of host-microbe cellular interactions. If this is the case, one can imagine that control of the post-mitotic intracellular particle distribution is critical area of contention of host and microbial cells with the emergence of microbial and host defense mechanisms to influence this outcome. In our system, we observed that Outcome I events were associated with a higher likelihood of phagosomal extrusion, and event that theoretically benefits the microbe by allowing fungal escape from a phagocytic cell. Since phagosomal extrusion is associated with the appearance of pathological changes in the host cell [28], we infer, that on balance, Outcome I benefits the *C. neoformans*. However, it is likely that the relative gains and debits of Outcome I and II events are system dependent and vary for individual microbe-host cell interactions.

Our results show that Outcome I events are far more common that would be expected by chance alone, thus implying the existence of mechanisms that preferentially sort microbial cargo into one daughter cell. Given that the probability of Outcome I drops rapidly with increasing cargo number, Outcome I events require either ingestion of very few microbes or phagosomal fusion. Since limiting phagocytosis in an infected area would seem counterproductive for both host defense and intracellular pathogens that thrive inside cells, it would appear that phagosomal fusion is the most effective mechanism for influencing microbial cargo distribution following phagocytic cell division. Inspection of the data obtained with live and dead, wild type and PLB1 mutant, encapsulated and non-encapsulated yeasts and inert polystyrene beads suggest that both the microbial and phagocytic cells can influence the distribution of ingested particle cargo following cell division. For polystyrene beads, the distribution of ingested particles was stochastic. Since polystyrene beads are inert, the absence of Outcome I events could reflect a lack of reaction by the host cell to trigger cellular mechanisms that would promote phagosome fusion. In contrast, for yeasts, the outcome of intracellular particle sorting into daughter cells was stochastic for strain 24067 and non-stochastic for acapsular *C. neoformans* strain Cap67, PLB1 mutant and *C. gattii*.

Giant phagosome formation was highly correlated with Outcome I events. In this system, phagosomal fusion is known to occur resulting in the formation of very large phagosomes [26], [28]. The mechanism of phagosomal fusion in *C. neoformans* infected cells has not been elucidated, and dissection of the microbial and host factors responsible for phagosomal fusion is beyond the scope of this study. A likely microbial candidate to influence phagosomal fusion is the capsular polysaccharide given that polymers such as polyethylene glycol promote membrane fusion and polysaccharides such as dextran produce vesicle aggregation [31]. However, the effect of polysaccharide appears to be variable and dependent on the cell. When exogenous polysaccharide was added to the acapsular Cap67 cells we noted a decrease in Outcome I events possibly reflecting disaggregation of clumped cells as a consequence of increased surface charge [32]. Phospholipase could also affect phagosomal fusion through effects on integrity of phagosomal membranes [33]. The secreted phospholipase B was documented to play a role in cryptococcal dissemination [34].

In the course of this study we noted two phenomena that appeared related to the occurrence, timing and efficacy of phagosomal fusion events. First, phagosomal extrusion events commonly occurred before or after cell division of macrophage-like cells, and this phenomenon was most commonly observed for phagocytic cells harboring *C. gattii.* The observation that host cell division can follow phagosomal extrusion provides solid evidence that macrophage-like cells are not significantly damaged by the extrusion process. Interestingly, we noted that most of extruded phagosomes with *C. gattii* remained attached to the macrophage-like cells undergoing cell division, consistent with the occurrence of altered membranes in these cells. The observation that phagosomal extrusion sometimes preceded cell division is intriguing because it suggests that this phenomenon may reflect a need for phagosomes to fuse before being extruded. Phagosomal extrusion with replication produces two uninfected phagocytic cells. Second, we noted that macrophage-like cells harboring yeasts occasionally fused after cell division. This observation is intriguing given that giant cell formation is thought to be critical for granuloma formation and containment of *C. neoformans* infection in tissue [35]. We are not sure whether this process is analogous to that which occurs *in vivo* but we note with interest that cell fusion events were limited to yeast infected cells. The finding that the capsular polysaccharide is involved in phagosomal extrusion [26] suggests a mechanism whereby the polysaccharide alters the cell and phagosomal membranes to make them more likely to fuse, like other pro-fusion molecules as polyethylene glycol. Consequently, macrophage-like cell fusion events may reflect contact between two cells with altered membranes.

In summary, we report that replication of phagocytic cells carrying a cargo of yeasts can produce both stochastic and non-stochastic outcomes with respect to intracellular yeast cell distribution into daughter cells depending on the yeast strains and species. This observation combined with the fact that mitotic events for cells carrying polystyrene beads produced stochastic outcomes implies that the distribution outcome is influenced by microbial factors. These results provide the first systematic analysis of particle distribution after phagocytic cell division and highlight the need to consider distribution outcome in evaluating the pathogenesis of infection. Microbial cargo distribution among daughter host cells may be as important a consideration as microbial intracellular location, mechanism of intracellular survival, and cellular exit strategy in influencing the outcome of infection. Consequently, we propose that the evaluation of microbial cargo distribution following phagocytic cell division is a new variable to be considered in studies of microbial pathogenesis and our findings suggest the need for similar studies with other intracellular pathogens.

## Materials and Methods

### Yeast strains and culture

*C. neoformans* var. *neoformans* strain 24067 and Cap67 (acapsular strain) was obtained from the American Type Culture Collection (Rockville, MD). *C. neoformans* var. *grubii* strain H99 and its phospholipase (PLB1) mutant were obtained from John Perfect (Durham, NC). PLB1 was documented as a virulence factor of *C. neoformans* [36]. *C. gattii* (Serotype B) strain NIH 198 was provided by Thomas Mitchell (Durham, NC). All yeasts were cultured in Sabouraud dextrose broth (Difco) at 30°C with agitation (150–180 rpm). When necessary, yeasts were killed by heating to 56°C in water bath for 1 h and yeast death was confirmed by demonstrating no colony growth in Sabouraud agar. To coat polysaccharide to acapsular Cap67 cells, *C. neoformans* strain H99 cultures were grown in Sabouraud dextrose broth for more than 3 days and supernatant was collected and filtered by 0.45 mm filter. Acapsular Cap67 yeast cells were incubated with filtered supernatant for 2 h. The presence of surface polysaccharide was established by indirect immunofluorescence [37], [38].

### Murine macrophages and culture

The murine macrophage-like J774.16 cells, originally derived from a reticulum sarcoma [39], were used for all the experiments except for those specifically indicated. J774.16 cells were selected because *C. neoformans* infection in this host cell replicates all the phenomena observed with primary cells [22], [26], [40]. J774.16 cells were grown at 37°C with 10% CO_2_ in feeding media consisting of Dulbecco's minimal essential medium (DMEM) (Life Technologies), 10% NCTC-109 medium (Gibco), 10% heat-inactivated (56°C for 30 min) FCS (Gemini Bio-products, Woodland, CA), and 1% non-essential amino acids (Mediatech Cellgro, Washington, DC). Cells were then plated on polylysine coverslip-bottom MatTek plates (MatTek Cultureware, Ashland, MA) at a density of 5×10^4^ per plate in feeding media, stimulated with 50 U/ml recombinant murine IFNγ (Genzyme, Cambridge, MA) and incubated overnight at 37°C and 10% CO_2_.

For primary murine macrophage harvesting, 6–8 week old BALB/c female mice (Jackson Laboratories, Bar Harbor, ME) were sacrificed by asphyxiation with CO_2_. Macrophages were harvested by lavaging the peritoneal cavity with sterile PBS (10 ml washes per mice). Cells were pooled and spun down at 1200 rpm, counted, resuspended in feeding media aforementioned and plated in MatTek plates. Macrophages were allowed to adhere for at least 1 h prior to phagocytosis and subsequent microscopic time-lapse imaging.

### Macrophage-like phagocytosis assay and time-lapse microscopy

For live cell imaging, 5×10^4^ macrophage-like cells were plated on polylysine coated coverslip bottom MatTek plates and allowed to adhere overnight as described above. Yeast cells or polystyrene beads (3.2 µm, Spherotech, Lake Forest, IL) were washed with PBS for 3 times prior to phagocytosis assays. The macrophage culture media was then removed and replaced with fresh media containing yeast cells at effector to target ratio of 10∶1 or 5∶1, or polystyrene beads at effector to target ratio of 10∶1 along with 10–50 µg/ml of purified anti-capsular mAb 18B7 when *C. neoformans* strain 24067 and *C. gattii* were used or 20% guinea pig serum when H99 strain was used. In comparing *C. neoformans* strains we faced the problem that there is great variability in the efficiency of antibody- and complement-mediated phagocytosis. For example, H99 is easily opsonized with complement while antibody is significantly less effective. Since the mode of opsonization does not appear to have major effect on the outcome of the fungal-macrophage interaction with respect to intracellular replication or exocytosis [19], [26], [41] and we were interested in cargo distribution after replication, we opted for using the most efficient opsonin for each strain. In all phagocytosis experiments, macrophage-like cells were activated with 0.3 µg/ml lipopolysaccharide (LPS) (Sigma, St. Louis, MO) and 50 units/ml of murine IFNγ. Macrophage-like cells and *C. neoformans* or beads were incubated together for 60 min to allow for completion of phagocytosis, washed once with fresh media, replenished with 2 ml feeding media and followed by time-lapse imaging every 4 minutes. Images were collected at 10× or 20× using the Axiovert 200 M inverted microscope and photographed with an AxiocamMR camera controlled by the Axio Vision 4.4 software (Carl Zeiss Micro Imaging, NY). This microscope was housed in a Plexiglas box and the temperature was stabilized at 37°C with a forced air heater system. The plate lid was kept in place to prevent evaporation, and 5% CO_2_ was delivered to a chamber locally at the culture dish. Images were compiled into movies which were then used to analyze the intracellular particle distribution and phagosome formation during macrophage-like cell division. Movie animations and montages were created using ImageJ software (http://rsb.info.nih.gov/ij/).

### Statistical analysis

One-way ANOVA, Newman-Keuls multiple comparison test, one sample *t* test, χ^2^ analysis and linear regression were used as indicated. Statistical analysis was done by Prism 4.0 (GraphPad Software, San Diego, CA) and *p*<0.05 was considered to be significant.

## Supporting Information

Movie S1 Movie S1 showed one post-mitotic Outcome I event of intracellular particle distribution.
(1.74 MB AVI)

Movie S2 Movie S2 showed one post-mitotic Outcome II event of intracellular particle distribution.
(4.55 MB AVI)

Movie S3 Movie S3 showed one fusion event of two daughter macrophage cells with intracellular yeasts post mitosis.
(0.24 MB AVI)

Movie S4 Movie S4 showed one extrusion event of intracellular yeasts during mitosis of macrophage-like cell.
(3.48 MB AVI)

Data Set S1 Data Set S1 was the java program for calculation of DI_T_.
(0.01 MB JAVA)

Data Set S2 Data Set S2 was the java program for calculation of P.
(0.01 MB JAVA)

Data Set S3 Data Set S3 was the java program for factorial calculation.
(0.01 MB JAVA)

Data Set S4 Data Set S4 was the calculated results of DI_T_.
(0.02 MB XLS)

Data Set S5 Data Set S5 was the calculated results of P.
(0.03 MB XLS)
